# Atypical presentation of COVID‐19 infection as acute abdomen in children: A case series

**DOI:** 10.1002/ccr3.5030

**Published:** 2021-11-06

**Authors:** Ali Khakshour, Amin Saeidinia, Ghazale Ghanbari

**Affiliations:** ^1^ Faculty of Medicine Mashhad University of Medical Sciences Mashhad Iran; ^2^ Faculty of Medicine Mashhad University of Medical Sciences Mashhad Iran; ^3^ Akbar Hospital Mashhad University of Medical Sciences Mashhad Iran

**Keywords:** Acute Abdomen, COVID19, Gastrointestinal presentation, Pediatrics

## Abstract

Abdominal pain in children in COVID‐19 era should be carefully evaluated and weighing risk‐benefit of surgical procedure in suspected cases should be more discussed. Surgery in patients with high suspicious to MIS‐C should be delayed for preventing critical outcomes.

## Graphical abstract

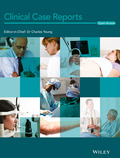

## 1 INTRODUCTION

Gastrointestinal symptoms have not been recognized in the early stages of the pandemic and are infrequently reported in the literature in COVID‐19 infection. We here reported two children with acute abdomen as an atypical presentation of COVID‐19 infection. Owing to the spread of the coronavirus disease 2019 (COVID‐19), new data on the pathologies of the disease have been emerged. However, reports on infection in children are still scarce.^1^ Children might have milder clinical manifestations than adults.^2^ It has been found that asymptomatic illness spreads through children.^3^

To date, majority of the cases and deaths have been reported in the adult population. Currently, concern regarding an inflammatory syndrome related to COVID‐19 in children with gastrointestinal symptoms with PCR tests showing the result of both positive and negative for SARS‐CoV‐2 has been reported.^4^ Gastrointestinal symptoms are infrequently reported in the literature in adults with COVID‐19 infection,^5^ but a high‐mean viral load in the nasopharynx has been associated with diarrhea in patients with severe acute respiratory syndrome.^6^, ^7^

We here reported two children with acute surgical abdomen as an atypical presentation of COVID‐19 infection.

## 2 CASE PRESENTATION

### 2.1 Case 1

A 12‐year‐old boy was presented with abdominal pain and vomiting for 5 days. Vomiting was bilious and progressive, and no response to outpatient (hyoscine and cefixime) treatment was achieved. Other symptoms included fever, anorexia, diarrhea, abdominal pain, numbness of extremities, photosensitivity, hyperacusis, and dysuria. The patient had no history of any particular disease. Vital signs on admission were defined as follows: BP, 110/70; T, 37.6; PR, 80; RR, 20; and SPO2, 98.

**FIGURE 1:**
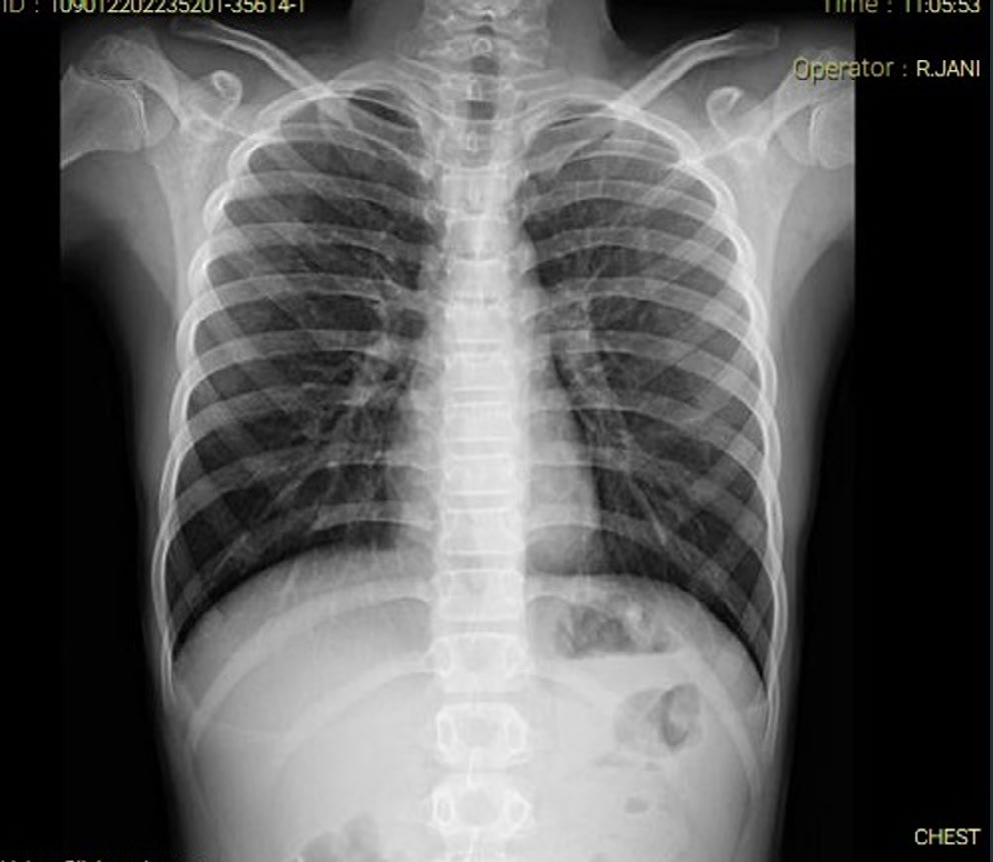
Chest X‐ray

**FIGURE 2:**
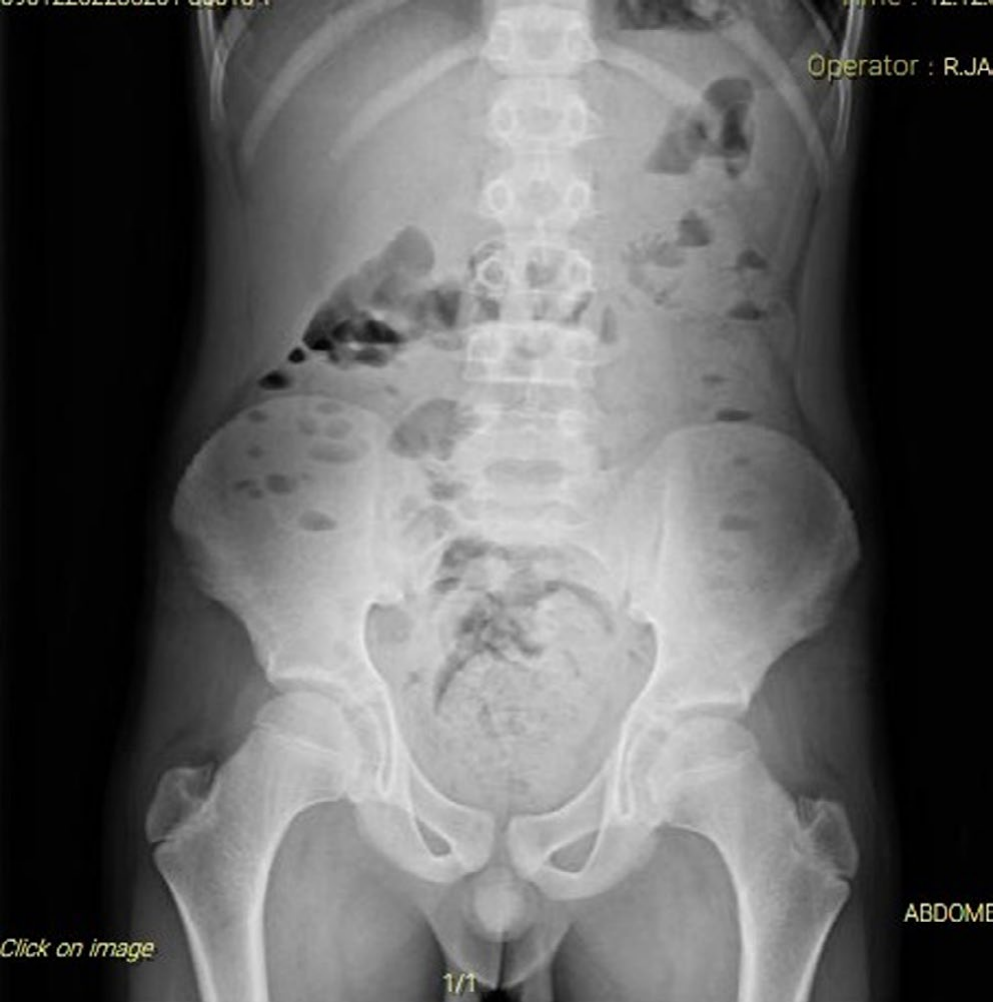
Abdominal X‐ray

He did not have abdominal distention, tenderness, or guarding. He had no respiratory symptoms. Chest and abdominal X‐ray was found normal (Figures 1 and 2). Lymphadenitis was reported in the abdominal ultrasound. Consultation for pediatric surgery was sought, which was not recommended.

Considering COVID‐19 pandemic, a nasopharyngeal sample was taken to test for SARS‐CoV‐2 and was found positive. Patient received conservative treatment and discharged after signs and symptoms resolved.

### 2.2 Case 2

A 5‐year‐old boy was presented with fever and vomiting for 4 days. His vomiting was bilious and progressive and did not respond to outpatient treatment. During the physical examination, left eye swelling suspicious of orbital cellulitis was detected, which was probably due to insect bite. A generalized tenderness with dominance in the epigastrium was detected. He had no meningismus stigmata during the examination, and the Kernig and Brudzinski signs had negative results. On ophthalmological examination, there was no papilledema. During hospitalization, he developed recurrent vomiting and abdominal pain with right lower quadrant tenderness resembling an acute appendicitis.

Abdominal ultrasound showed no signs for appendicitis, and there was mesenteric lymphadenitis. Chest X‐ray had normal findings. Consultation for pediatric surgery was requested but was not recommended.

No pathologic findings were reported in the laboratory data. Due to COVID‐19 pandemic and for the possibility of atypical gastrointestinal presentations, a nasopharyngeal sample was taken to test for SARS‐CoV‐2, which was negative.

After the required treatment ciprofloxacin ophthalmic drop for cellulitis and oral clindamycin capsule, his symptoms diminished and was discharged. In the follow‐up, SARS‐CoV‐2 antibodies were checked using SARS‐CoV‐2 IgM ELISA kits (Pishtaz Teb, Iran, http://pishtazteb.com) and SARS‐CoV‐2 IgG ELISA kits (Pishtaz Teb, Iran http://pishtazteb.com) according to the manufacturer's protocol. IgG and IgM antibodies had shown positive results in the patient and in his parents.

## 3 DISCUSSION

The most prevalent symptom of COVID‐19 infection in adults is respiratory disease, while in children, it is not specific.^8^ COVID‐19 patients might present with other clinical manifestations such as gastrointestinal complaints, but they are uncommon.^8^ Abdominal pain in adults and children due to SARS‐CoV‐2 infection is an unusual clinical sign.^9^, ^10^

In this case series, we presented two children with acute abdomen linked with COVID‐19 infection. In a case series, five children with COVID‐19 infection were presented with acute abdomen.^10^ None of patients underwent an operation but were admitted in PICU for ruling out MIS‐C (multisystem inflammatory syndrome in children) related to COVID‐19. All of them were treated medically and were discharged with a good condition. In another study, four patients were reported with acute abdomen; of them, one underwent a surgical treatment^8^ and the others were treated medically with good outcomes. In our study, both of our patients were febrile and had bilious vomiting, which were non‐specific for COVID‐19 infection.

In our case study, fever and vomiting were predominant. In another study, all five patients had fever and abdominal pain: 60% presented with vomiting and diarrhea (simultaneously), and the rest had only vomiting.^10^ Another study reported eight children at a single center in the UK with symptoms of atypical appendicitis. The most common gastrointestinal symptoms were abdominal pain, diarrhea, and vomiting.^11^

CT scan or abdominal sonography can be useful to detect the cause of abdominal pain in some cases. It was shown that abdominal ultrasonography in COVID‐19 patients was compatible with terminal ileitis.^10^ Our cases had non‐specific findings in abdominal imaging. In our study, both patients did not undergo surgical intervention and were treated conservatively. The evaluation of patients with abdominal pain during COVID‐19 pandemic should be performed cautiously to distinguish other conditions. An unnecessary surgery in such patients can lead to a cytokine storm and deterioration of patients’ condition.^12^ Therefore, a decision for surgical approach should be taken with much caution and conservative treatment might be considered on admission. A multidisciplinary decision is necessary, and laboratory and imaging findings would help for a proper diagnosis and management.

## 4 CONCLUSIONS

In contrast to adult patients, children could present with different manifestations, such as abdominal pain. We here reported two children with acute abdomen mimicking a surgical abdomen. Therefore, pediatricians need to distinguish GI symptoms from COVID‐19 presentations.

## CONFLICT OF INTEREST

There is no conflict of interest. We declare that none of the authors listed on the manuscript are employed by a government agency that has a primary function other than research and/or education. None of the authors are submitting this manuscript as an official representative or on behalf of the government.

## AUTHOR CONTRIBUTIONS

Dr. Ali Khakshour and Dr. Amin Saeidinia managed patients and wrote the first draft of the manuscript, and Miss. Ghazale Ghanbari collected patients’ data and wrote the final version. All authors read and approved final version.

## CONSENT

The authors confirmed that the signed consent was obtained from patients in accordance with the journal's patient consent policy.

## Data Availability

The data that support the findings of this study are available from the corresponding author upon reasonable request.
